# Supplementary material for: Gfi1 Loss Protects against Two Models of Induced Diabetes
Source: Cells. 2021 Oct 20;10(11):2805. doi: 10.3390/cells10112805 (PMC8616283; doi:10.3390/cells10112805)
Supplement: Supplementary file 1 [file cells-10-02805-s001.zip › cells-1393651-supplementary/cells-1393651-sp1019/cells-1393651-sp1019.pdf]

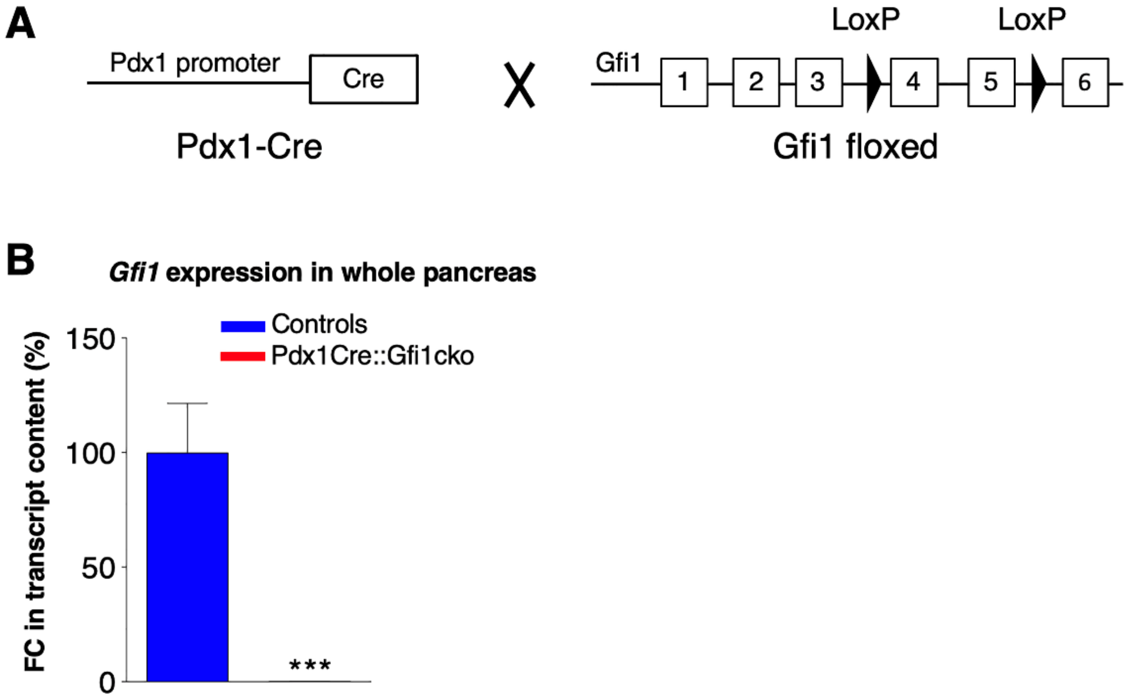

Figure S1. Generation of Pdx1Cre::Gfi1cko mouse line. To specifically inhibit *Gfi1* expression in the pancreas, Gfi1Cre animals (harboring a transgene composed of *Cre recombinase* downstream the *Pdx1* promoter) were crossed with Gfi1cko animals (in which the 4th and 5th exon of the *Gfi1* gene are flanked by two *LoxP* sites). In the resulting Pdx1Cre::Gfi1cko mice (**A**), Cre recombinase mediates the irreversible excision of *Gfi1 DNA binding domain* in all the pancreatic cells starting from the onset of pancreas specification. Cre recombinase efficiency was verified by qPCR (*n* = 3 animals per genotype) (**B**).

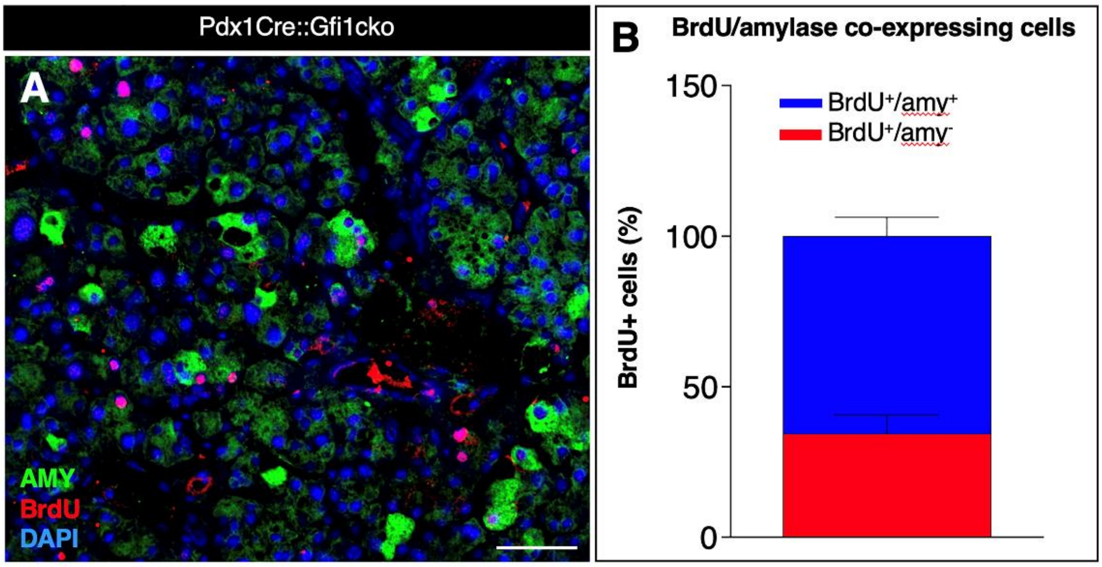

Figure S2. Proliferative cells in pancreatic acinar compartment of Pdx1Cre::Gfi1cko mice are predominantly negative for amylase immunostaining. Pancreatic sections of *Gfi1*-lacking mice were stained using antibodies specifically recognizing BrdU and amylase. Objective magnification: 20X. Scale bar: 50µm (**A**). Quantification of BrdU+ acinar cells co-expressing or not amylase in *Gfi1*-mutant pancreatic tissue. (*n* = 86 BrdU+ cells) (**B**).

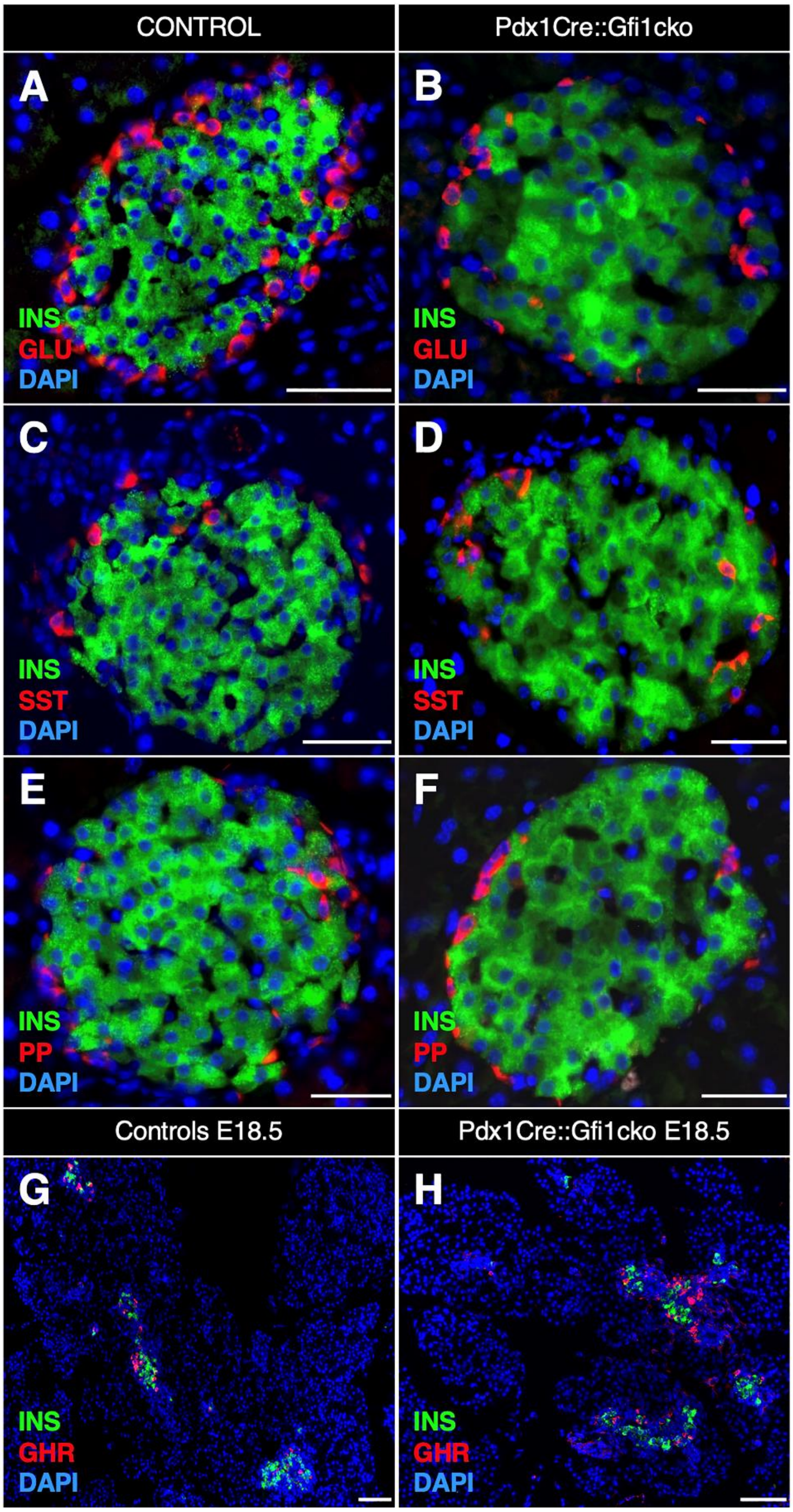

Figure S3. Loss of *Gfi1* does not perturb islets of Langerhans architecture. Immunohistochemical analyses for insulin (**A**–**F**), glucagon (**A**–**B**), somatostatin (**C**–**D**) and pancreatic polypeptide (**E**–**F**) of pancreatic sections isolated from Pdx1Cre::Gfi1cko adult animals and controls. Objective magnification: 20X. Scale bar: 50μm. Immunofluorescent identification of ghrelin-expressing cells at E18.5, in control and *Gfi1*-mutant pancreatic sections. Objective magnification: 10X. Scale bar: 100 μm (**G**–**H**).

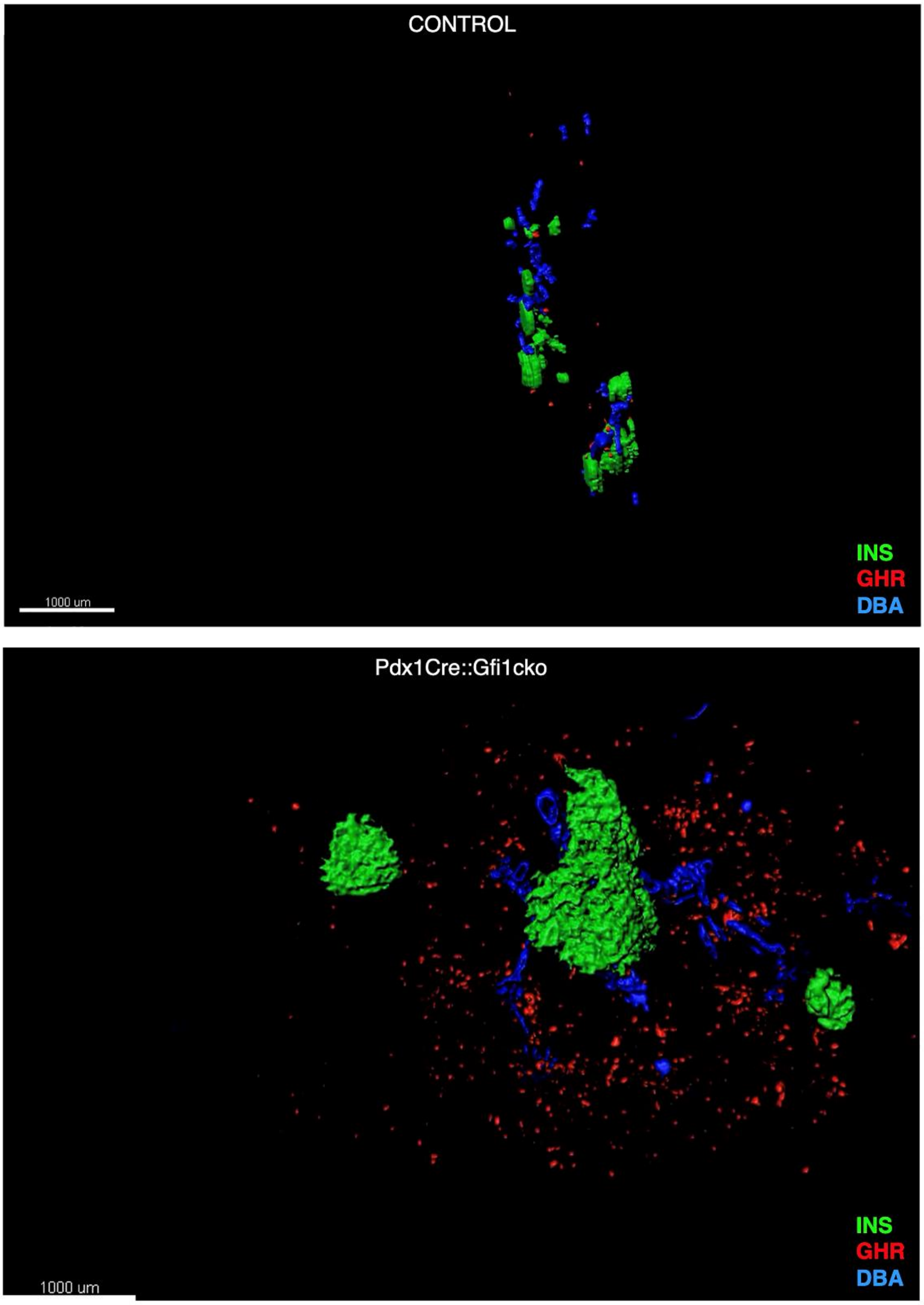

Figure S4. Extract of Movie S1. Ghrelin is massively expressed in adult *Gfi1*-deficient acinar cells.

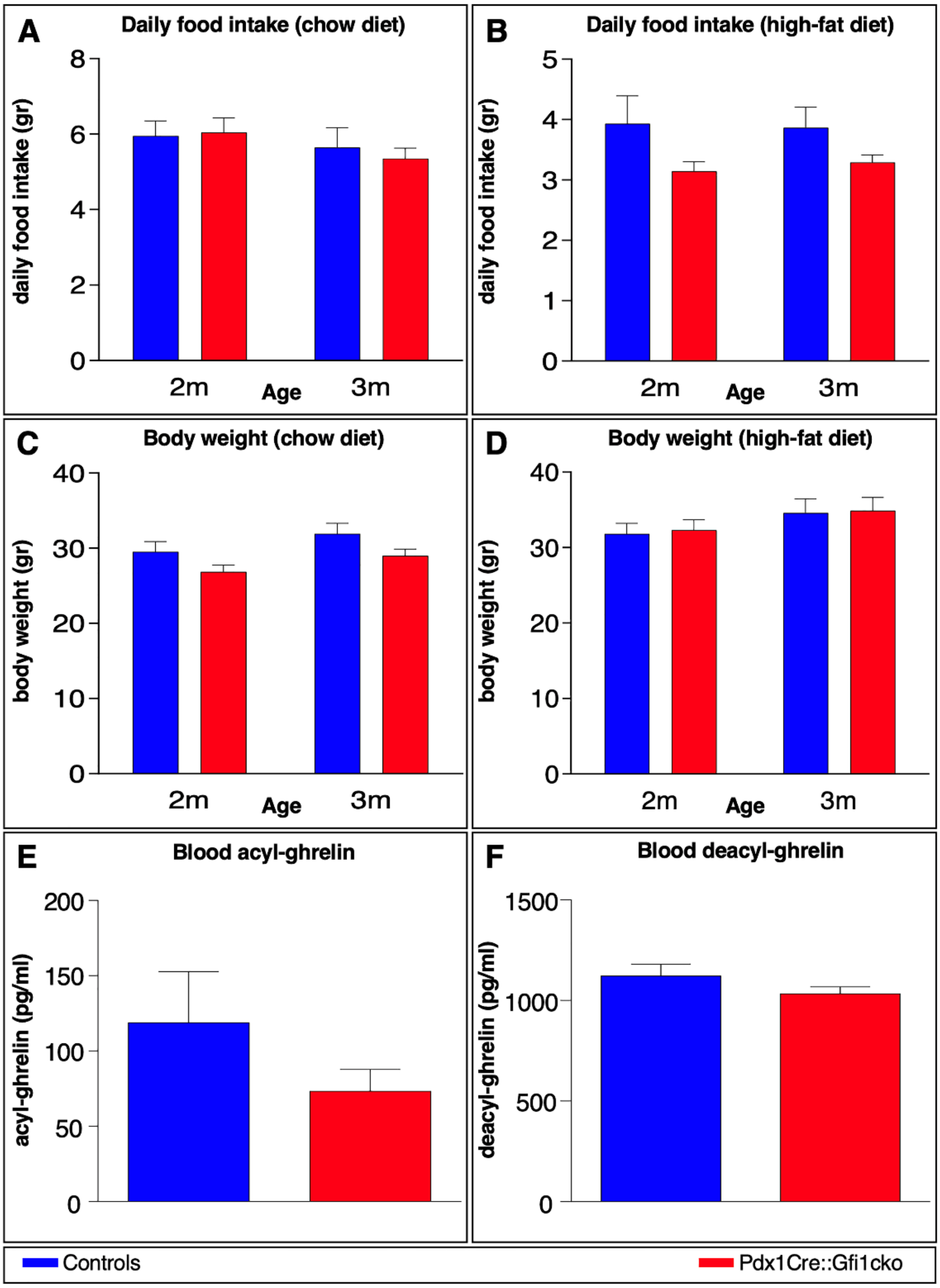

Figure S5. Increase in intrapancreatic ghrelin in Pdx1Cre::Gfi1cko animals does not affect food intake, body weight nor ghrelinemia. Daily food intake of adult Pdx1Cre::Gfi1cko and age-matched control mice fed with either chow or HFCD (n = 7 animals per genotype) (**A**–**B**). Body weight of adult control and *Gfi1*-mutant mice under chow or HFCD regimen (n = 7 animals per genotype) (**C**–**D**). ELISA measurement of blood acyl-ghrelin (**E**) and deacyl-ghrelin (**F**) (n = 6 control and n=8 Pdx1Cre::Gfi1cko samples).

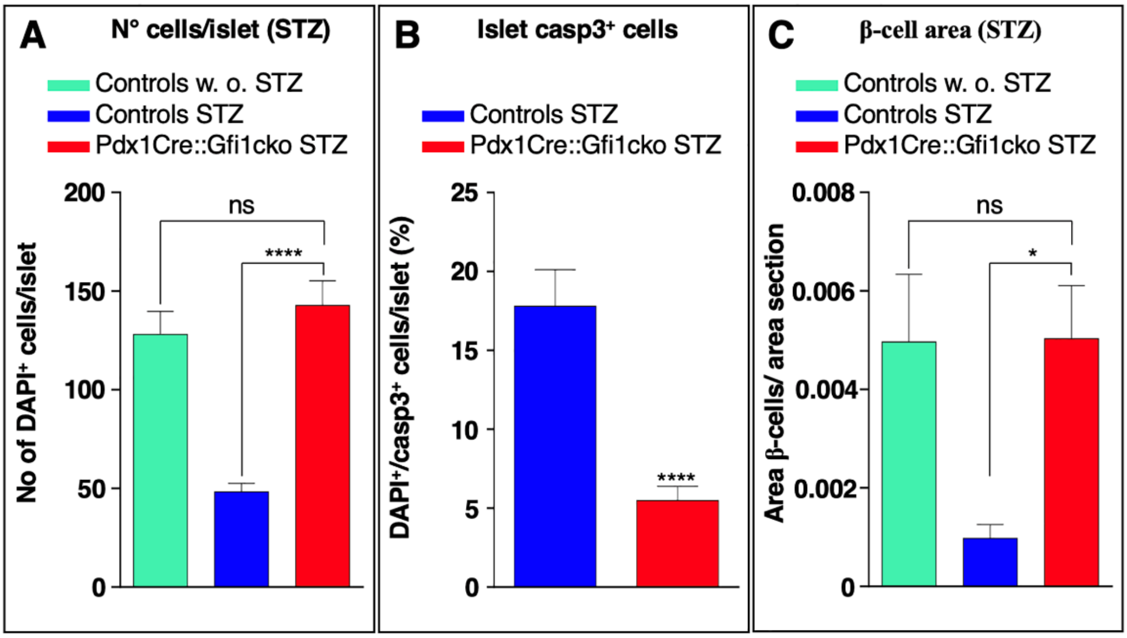

Figure S6. STZ exerts negligible apoptotic effects on β-cells of Pdx1Cre::Gfi1cko mice. Pdx1Cre::Gfi1cko and control animals were injected or not with high doses of STZ and the number of cells per islet was assessed 24 h later. (*n* = 72 islets for control w. o. STZ; *n* = 49 islets for control STZ; *n* = 79 islets for Pdx1Cre::Gfi1cko STZ) (**A**). Evaluation of the percentage of islet cells positive for caspase-3 immunostaining in *Gfi1* loss-of-function animals as compared to control upon STZ treatment (*n* = 49 islets for control STZ; *n* = 79 islets for Pdx1Cre::Gfi1cko STZ) (**B**). Quantification analyses of insulin immunolabeled cells of control and Pdx1Cre::Gfi1cko animals one week following STZ challenge and control mice not injected with STZ (*n* = 4 animals per condition) (**C**).

Movie Legend: Movie S1. Pdx1Cre::Gfi1cko pancreatic acinar cells ectopically express ghrelin during adulthood. Pancreata (*n* = 3) from 3-months old control (**A**) and Pdx1Cre::Gfi1cko animals (**B**) were sectioned (8 µm thickness). 25 consecutives sections were stained with fluorescent DBA (blue), anti-ghrelin (red), and anti-insulin (green). Representative islets of Langerhans were photographed on each section, and the islet contour was manually laid out according to DAPI counterstaining. Using Amira software (Mercury), the pictures were aligned to form a z-stack, and movies of the resulting isosurface representation were generated. Note the striking abundance of ghrelin-expressing cells within the acinar compartment of Pdx1Cre::Gficko pancreata.
